# Reduced Granulosa Cell AKT2 mRNA Expression in Women with Unexplained Infertility: A Prospective Case–Control Study of ICSI Cycles

**DOI:** 10.3390/biomedicines14092108

**Published:** 2026-09-18

**Authors:** Özgür Uzun, Belgin Devranoğlu, Enis Özkaya, Huri Demirci, Gülden Tunalı, Zehra Sezer, Fatma Beyza Sağ, Emine Elif Güzel Meydanlı

**Affiliations:** 1 Department of Histology and Embryology, Cerrahpaşa Faculty of Medicine, Istanbul University-Cerrahpaşa, Istanbul 34098, Türkiye; 2 Assisted Reproductive Technology Unit, Department of Obstetrics and Gynecology, Zeynep Kâmil Women and Children’s Diseases Training and Research Hospital, University of Health Sciences, Istanbul 34668, Türkiye; 3 Department of Medical Biochemistry, Faculty of Medicine, Biruni University, Istanbul 34010, Türkiye

**Keywords:** unexplained infertility, AKT2, PI3K/Akt signaling, granulosa cells, cumulus cells, follicular fluid, oocyte competence, ICSI

## Abstract

**Background/Objectives**: Unexplained infertility (UI) is defined by the absence of detectable abnormality on standard evaluation, which raises the possibility that the causative defect lies at the level of intracellular signaling rather than at the level of gamete number or hormone concentration. The PI3K/Akt pathway governs primordial follicle activation, granulosa cell survival, and the acquisition of oocyte competence, but AKT2 transcript abundance in the somatic follicular compartment has not been characterized clinically in UI. We compared cumulus/granulosa cell AKT2 transcript levels and follicular fluid Akt and phospho-Akt (Ser473) protein levels between women with UI and women undergoing intracytoplasmic sperm injection (ICSI) for isolated male factor infertility.  **Methods**: This was a prospective case–control study of 128 women (64 UI, 64 male factor) aged 23–35 years, all stimulated with a GnRH antagonist protocol and recruited December 2020–May 2021. AKT2 expression in cumulus/granulosa cells recovered at denudation was quantified using RT-qPCR (ΔΔCt, GAPDH reference). Total Akt and phospho-Akt (Ser473) were measured in follicular fluid using ELISA and normalized to the number of oocytes retrieved. Oocyte maturation, fertilization, day 3 embryo quality, and day 5 blastocyst quality were assessed using Alpha/ESHRE Istanbul consensus criteria. Correlation analyses were exploratory and reported with Benjamini–Hochberg false discovery rate (FDR) adjustment. **Results**: Baseline characteristics, including age, body mass index, antral follicle count, AMH, FSH, LH, oocytes retrieved, and duration of infertility, were comparable between groups. Granulosa cell AKT2 ΔCt was higher in the UI group (3.46 ± 1.47 versus 2.68 ± 1.18; *p* = 0.001), corresponding to a 1.72-fold lower AKT2 transcript level (ΔΔCt 0.78; Cohen’s d = 0.59); the difference persisted after adjustment for age, body mass index, AMH, basal estradiol levels, and previous IVF attempts (adjusted ΔCt difference 0.74, 95% CI 0.25–1.24; *p* = 0.004). Follicular fluid total Akt (11.55 ± 4.08 versus 11.75 ± 3.60 relative units; *p* = 0.78) and phospho-Akt (21.05 ± 7.14 versus 19.81 ± 7.24; *p* = 0.34) did not differ, nor did values normalized per oocyte. Oocyte maturation rate (0.72 ± 0.25 versus 0.76 ± 0.18), fertilization rate (44.6 ± 29.5% versus 42.2 ± 25.6%), day 3 good-quality embryo rate (25.0 ± 25.8% versus 26.6 ± 23.8%), and good-quality blastocyst rate (30.2 ± 25.8% versus 20.5 ± 18.5%) were all comparable. Of 24 exploratory correlations, only two survived FDR adjustment. Both correlations were in the male factor comparison group and both involved the small blastocyst subgroup: total Akt per oocyte (ρ = 0.738) and phospho-Akt per oocyte (ρ = 0.728) versus good-quality blastocyst rate (*n* = 17; FDR *p* = 0.011). AKT2 ΔCt did not correlate with any embryological endpoint in either group. **Conclusions**: Cumulus and granulosa cell AKT2 mRNA was reduced approximately 1.7-fold in women with unexplained infertility, despite indistinguishable ovarian reserve, ovarian response, and embryological performance and despite unchanged follicular fluid Akt protein. Because the comparison group consisted of women from couples undergoing ICSI for male factor infertility rather than women of proven fertility, the difference cannot be attributed specifically to unexplained infertility, and the findings describe transcript abundance rather than pathway activity. Confirmation against a fertile comparison group, with protein-level assessment in the same cells and clinical pregnancy endpoints, is required.

## 1. Introduction

Unexplained infertility (UI) is a diagnosis of exclusion, assigned when semen analysis, documented ovulation, and tubal patency are all normal [1,2]. Despite decades of diagnostic refinement, it continues to account for a substantial share of infertile couples [3,4,5,6]. That persistence is informative: it suggests the routine work-up does not interrogate the biological level at which the defect resides. Standard testing measures follicle number and circulating hormone concentrations, not the signal transduction machinery that converts gonadotropin stimulation into oocyte developmental competence.

The phosphatidylinositol-3-kinase (PI3K)/Akt pathway is central to that machinery. In the oocyte, PI3K/Akt activity controls the transition of primordial follicles from quiescence to growth; oocyte-specific Pten deletion causes premature global activation and eventual exhaustion of the follicle pool [7,8,9]. In granulosa cells, the pathway mediates FSH-dependent survival and proliferation, suppresses apoptosis, and participates in the regulation of oocyte meiotic resumption [10,11]. Pathway defects have accordingly been linked to premature ovarian insufficiency and diminished ovarian reserve, yet the mechanisms translating PI3K/Akt abnormality into human reproductive failure remain undefined [8,10].

Recent syntheses have reinforced how tightly this pathway is tied to female fertility across the whole reproductive sequence. Contemporary reviews place PI3K/Akt at the center of primordial follicle activation, granulosa cell survival, and the acquisition of developmental competence and describe how its dysregulation contributes to polycystic ovary syndrome and premature ovarian insufficiency [12,13]. Somatic cells, rather than the oocyte alone, are now understood to initiate follicle activation in the adult ovary [14,15], which places the granulosa and cumulus compartment at the center of the question rather than at its periphery. Akt activity also rises through meiotic maturation and remains elevated through fertilization and early cleavage, and oxidative stress that suppresses PI3K/Akt signaling in granulosa cells has been linked to accelerated ovarian ageing and subfertility [13,16].

Cumulus and granulosa cells offer a practical route to this question. They are removed as a matter of routine at denudation and would otherwise be discarded, so sampling them adds neither risk to the patient nor an additional procedure to the cycle. In addition, their transcriptional state reflects the bidirectional communication with the oocyte they surround [11,17]. For this reason, cumulus transcripts have been pursued as non-invasive candidate markers of oocyte competence. The intracytoplasmic sperm injection setting adds a further methodological advantage. Specifically, fertilization does not depend on sperm–zona binding or on penetration of the cumulus mass, so the male factor comparison group is not disadvantaged at the fertilization step by the condition that defines it. In addition, differences observed between the groups are less readily attributed to the male gamete.

Among the pathway’s three Akt isoforms, *AKT2* is of particular interest in the ovarian somatic compartment, where it participates in insulin- and gonadotropin-responsive signaling and has been characterized in ovarian tissue [18]. If reduced *AKT2*-mediated signaling can compromise oocyte competence without altering follicle counts or serum hormone levels, the resulting clinical picture would be indistinguishable from unexplained infertility.

We therefore compared cumulus/granulosa cell AKT2 transcript levels and follicular fluid Akt and phospho-Akt protein levels between women with UI and women undergoing ICSI for isolated male factor infertility—a comparison group whose female partners have no identified infertility factor—and related these markers to oocyte maturation and early embryo development.

## 2. Materials and Methods

### 2.1. Study Design, Setting, and Ethics

This prospective case–control study was conducted in the Assisted Reproductive Technology Unit of Zeynep Kâmil Women and Children’s Diseases Training and Research Hospital, Istanbul, Türkiye, between December 2020 and May 2021. The protocol was approved by the Clinical Research Ethics Committee of Haydarpaşa Numune Training and Research Hospital (HNEAH-KAEK 2020/238; 9 November 2020) and conducted in accordance with the Declaration of Helsinki. All participants gave written informed consent. Every follicular fluid and cell sample was coded with an anonymized identifier, so laboratory analyses were performed blinded to group allocation and to embryological outcome. Reporting follows the STROBE recommendations for observational studies [19]; the completed checklist is provided as File S1.

Recruitment was consecutive over the study period, and all women meeting the eligibility criteria during that window were invited to participate. Group allocation followed the clinical diagnosis already established during the routine infertility work-up and was not modified for study purposes. Laboratory personnel handling the follicular fluid and cell samples received only the anonymized identifier; the assays were performed after the clinical cycles had been completed, so laboratory results could not have influenced embryological grading or any treatment decision.

### 2.2. Participants

Eligible participants were women aged 23–35 years undergoing IVF/ICSI. The UI group comprised women with unexplained infertility diagnosed by exclusion, consistent with the definition subsequently formalized in the ESHRE evidence-based guideline [6]; the male factor comparison group comprised women with a normal fertility evaluation undergoing ICSI for isolated male factor infertility. Only cycles stimulated with a GnRH antagonist protocol were included to standardize the effect of controlled ovarian stimulation. Exclusion criteria were age outside 23–35 years, previous ovarian surgery, relevant chronic systemic disease, and use of any stimulation protocol other than the antagonist protocol. The sample size was determined a priori: detecting a standardized between-group difference of Cohen’s d = 0.5 with a two-sided α = 0.05 and 80% power requires 63 women per group, and 64 were enrolled in each.

The choice of comparison group requires explicit justification. Women undergoing ICSI for isolated male factor infertility were selected because they undergo the same stimulation, retrieval, and laboratory procedures, which allows the two groups to be compared under identical clinical conditions and makes cumulus cell sampling ethically straightforward. They are not, however, women of proven fertility: they belong to infertile couples and may differ biologically from a fertile population in ways that were not measured here. Any difference observed between these two infertile populations therefore cannot be attributed specifically to unexplained infertility, and this group is referred to throughout as the male factor comparison group rather than as a control group.

### 2.3. Sample Collection

Transvaginal ultrasound-guided oocyte retrieval was performed 35 h after the ovulation trigger. Aspirate from follicles ≥18 mm was examined under a stereomicroscope. After cumulus–oocyte complexes were transferred to culture medium, the remaining follicular fluid was pooled per patient and centrifuged at 1500× *g* for 15 min, and 2 mL of supernatant was coded and stored at −80 °C. Because fluid was pooled per patient, protein values are reported both as measured concentrations and normalized to the number of oocytes retrieved.

The consequence of pooling should be stated explicitly. A single sample per patient describes the aggregate follicular environment of that cycle and cannot be attributed to any individual oocyte, which precludes oocyte-level analysis and means that a woman contributing twelve follicles and a woman contributing three are represented by measurements of different provenance. Normalization to the number of oocytes retrieved was applied to address this asymmetry, and both normalized and unnormalized values are reported throughout so that the effect of that choice on the results can be examined directly.

Cumulus and corona radiata cells removed by routine hyaluronidase denudation constituted the second sample type; an RNA stabilization reagent was applied and samples were stored at −80 °C. Denuded oocytes were assessed under an inverted microscope. Metaphase II (MII) oocytes were classified as mature and underwent ICSI, while metaphase I and germinal vesicle oocytes were classified as immature.

### 2.4. Embryological Assessment and Outcome Definitions

Fertilization was assessed 16–18 h after ICSI according to the Alpha/ESHRE Istanbul consensus [17]. Embryos were cultured at 37 °C in 5% CO_2_. Day 3 embryos were graded by blastomere number, symmetry, and fragmentation; only embryos with seven or eight blastomeres, equal blastomere size, and grade 1 morphology (<10% fragmentation) were classified as good quality. Blastocysts were graded on day 5 [17,20], with 3AA, 4AA, 1AA, 2AA, 3AB, 4AB, 3BA, and 4BA classified as good quality [21].

Outcomes were the mature oocyte rate (MII/retrieved), fertilization rate (2PN/MII), day 3 good-quality embryo rate, and day 5 good-quality blastocyst rate. Clinical endpoints (implantation, clinical pregnancy, and live birth) were not available.

### 2.5. ELISA

Total Akt and phospho-Akt (Ser473) were measured in follicular fluid supernatant with a commercial sandwich ELISA (human Akt (pS473) + total Akt, Abcam, Cambridge, UK; ab126433) per the manufacturer’s instructions. Samples and kit positive controls were applied to anti-pan-Akt-coated microplates and incubated for 2.5 h; after washing, anti-phospho-Akt (Ser473) or anti-pan-Akt detection antibody was added for 1 h, followed by HRP-conjugated anti-rabbit IgG. TMB one-step substrate was added for 30 min in the dark. The reaction was stopped, and absorbance was read at 450 nm (Thermo Scientific microplate reader, Waltham, MA, USA). The assay detection range was 1.48–40 µg/mL with a sensitivity of 1.01 µg/mL; concentrations were derived from a linear standard curve. One phospho-Akt value (1.10 µg/mL) fell below the lowest standard and is reported as measured. Separate standard curves were constructed for the pS473 and pan-Akt assays. Because both curves are calibrated against the kit’s common Akt standard rather than against purified phospho- or total-Akt protein, the two readouts are reported as relative units on assay-specific scales and are not comparable to one another in absolute terms. Samples were assayed in single wells; intra- and inter-assay coefficients of variation could therefore not be determined.

### 2.6. RNA Isolation and AKT2 RT-qPCR

Total RNA was extracted from cumulus/granulosa cell pellets with the PureLink RNA Mini Kit (Thermo Fisher Scientific, Waltham, MA, USA; catalog no. 12183020) including on-column DNase I treatment. RNA purity was verified spectrophotometrically (A260/A280 ≈ 2.0; NanoDrop, Thermo Fisher Scientific, Waltham, MA, USA), and RNA was stored at −80 °C. cDNA was synthesized with the High-Capacity cDNA Reverse Transcription Kit and random hexamers (Applied Biosystems, Foster City, CA, USA; 4368814) with RNase inhibitor (N8080119).

Quantitative PCR was performed on a StepOnePlus Real-Time PCR System (Applied Biosystems, Foster City, CA, USA) using a commercial PI3K/AKT gene expression analysis kit (Hibrigen Biotechnology, Istanbul, Türkiye; catalog no. MG-AKT-01) according to the manufacturer’s instructions in 10 µL reactions containing 100 ng cDNA. Primer sequences supplied for the AKT2 and GAPDH assays are given in Table 1. Cycling was 95 °C/30 s; 40 cycles of 95 °C/15 s, 60 °C/30 s, and 60 °C/60 s; followed by melt curve analysis. All reactions were run in technical triplicate, and the mean Ct of the three replicates was used for ΔCt and ΔΔCt calculation. Relative AKT2 expression was normalized to GAPDH and calculated using the 2^−ΔΔCt^ method. Assay reporting follows the MIQE recommendations [22,23]. ΔCt was defined as Ct(AKT2) − Ct(GAPDH). Because ΔCt is inversely related to transcript abundance, a higher ΔCt denotes lower AKT2 expression.

Amplification efficiencies were 98.4% for *AKT2* and 100.5% for *GAPDH*, with standard curve R2 values of 0.997 and 0.998, respectively, satisfying the requirement of comparable target and reference efficiencies for the comparative Ct method. No-template controls were included in each run, and melt curve analysis was performed after amplification to confirm the specificity of the products. The manufacturer specifies an amplicon size of approximately 150 bp for both assays. Primer specificity was verified in silico against the NCBI Human Messenger RNA Reference Sequence database (Primer-BLAST, https://www.ncbi.nlm.nih.gov/tools/primer-blast/, accessed on 25 August 2026).

### 2.7. Statistical Analysis

Distributional assumptions were assessed with the Shapiro–Wilk test. Continuous variables are summarized as mean ± SD and, where distributions were skewed, additionally as median with interquartile range. Groups were compared with the independent samples *t*-test (Welch) and the Mann–Whitney U test, and both *p* values are reported so that the conclusion does not rest on the choice of test. The *AKT2* ΔCt comparison was prespecified as the primary analysis; the standardized mean difference (Cohen’s *d*) is reported alongside it.

Associations between PI3K/Akt markers and embryological endpoints were assessed using Spearman correlation. These 24 correlations were exploratory. Raw *p* values are reported together with Benjamini–Hochberg FDR-adjusted values, and only FDR-adjusted significance is interpreted. Two-sided *p* < 0.05 was considered significant. Analyses were performed in Python 3.12 (SciPy 1.14, statsmodels 0.14); the analysis script is available with the data on request.

Two features of this approach were deliberate. Welch’s form of the *t*-test was used rather than the equal variance form because group variances differed for several variables and the Welch test is the more conservative default when equality cannot be assumed. Reporting a parametric and a rank-based test for every comparison, rather than selecting one, was chosen because most distributions were skewed; presenting a single test would leave open the question of whether any conclusion depended on that selection. For the correlation analysis, the number of tests was fixed in advance by the design—three markers, four endpoints, two groups—so the Benjamini–Hochberg procedure could be applied across the complete family rather than to a subset chosen after inspecting the results.

Because the design is observational, the primary comparison was repeated in a multivariable linear regression with *AKT2* ΔCt as the dependent variable and group, age, body mass index, AMH, basal estradiol, and the number of previous IVF attempts as covariates. A sensitivity model substituted antral follicle count for AMH. Confidence intervals for the Spearman correlation coefficients were obtained using bias-corrected bootstrap resampling with 10,000 replicates.

## 3. Results

### 3.1. Cohort and Baseline Characteristics

**Figure 1:**
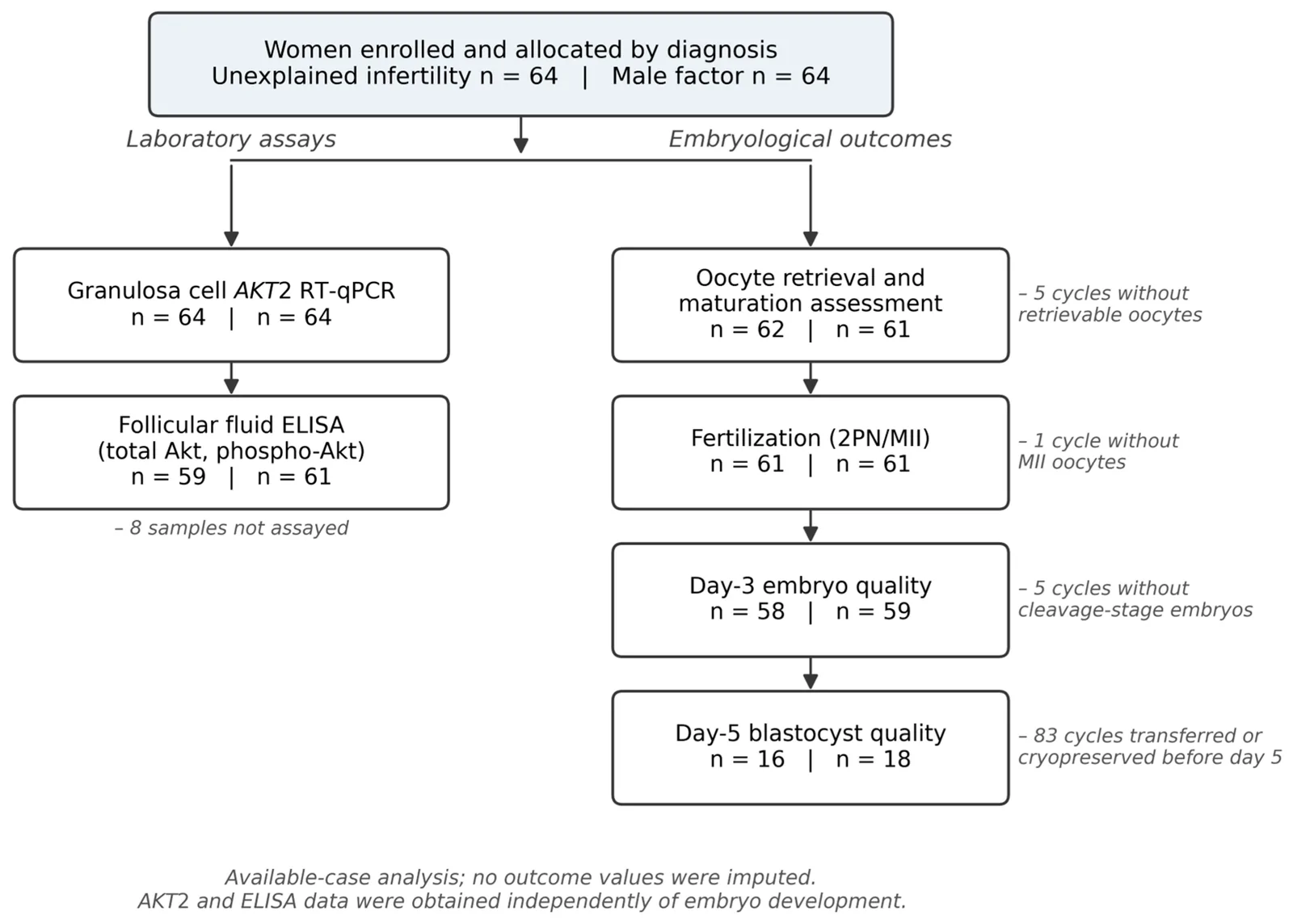
Study flow and availability of data at each stage. Laboratory assays (RT-qPCR and ELISA) and embryological outcomes were obtained independently; available case analysis was used throughout, and no outcome values were imputed.

Sixty-four women with UI and 64 forming the male factor comparison group were analyzed. Age, height, weight, body mass index, antral follicle count, duration of infertility, oocytes retrieved, FSH, LH, prolactin, TSH, and AMH did not differ between groups (Table 2). Two variables reached nominal significance: the number of previous IVF attempts was lower in the UI group (1.53 ± 1.04 versus 1.94 ± 1.22; *p* = 0.031), and basal estradiol was higher (54.37 ± 29.16 versus 44.84 ± 18.12 pg/mL; *p* = 0.014). Both differences are small in absolute terms and of doubtful clinical significance. The available case follow-up sequence is shown in Figure 1.

Shapiro–Wilk testing indicated departure from normality for most variables, including ovarian reserve markers, follicular fluid protein concentrations, and all embryological rates. Group comparisons are therefore reported with both the parametric and the rank-based test throughout Table 2 and Table 3, and the two approaches led to the same conclusion for every variable examined.

### 3.2. Granulosa Cell AKT2 Expression (Primary Analysis)

**Figure 2:**
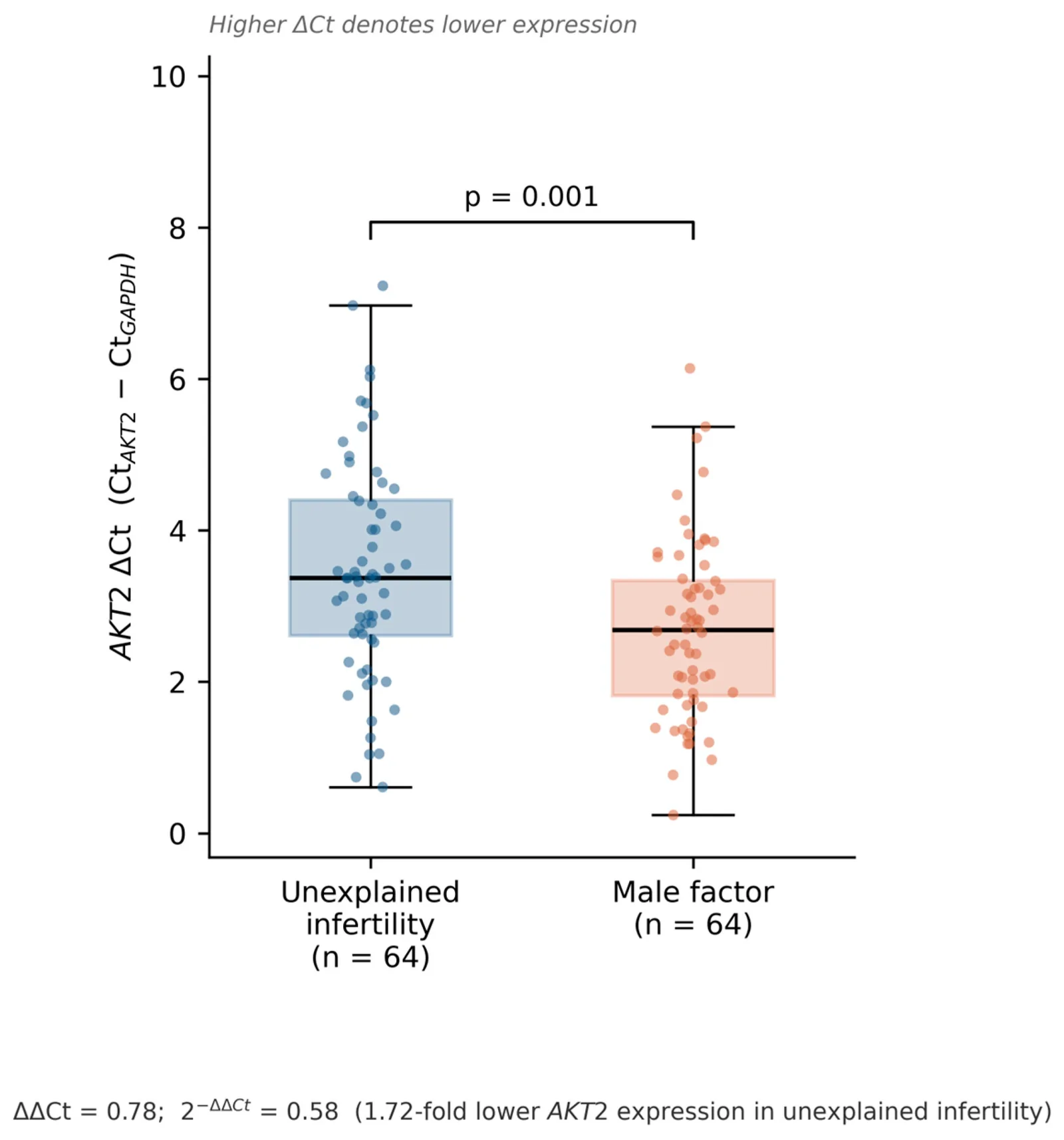
Cumulus/granulosa cell AKT2 ΔCt by group. Boxes show the median and interquartile range. Whiskers extend to 1.5 times the interquartile range, and points represent individual participants. ΔCt was calculated as Ct(AKT2) − Ct(GAPDH); a higher ΔCt denotes lower AKT2 expression. Comparison using Welch *t*-test.

Cumulus/granulosa cell AKT2 ΔCt was higher in the UI group than in the male factor comparison group (3.46 ± 1.47 versus 2.68 ± 1.18; median 3.37 [2.61–4.41] versus 2.69 [1.82–3.34]; *t*-test *p* = 0.0011, Mann–Whitney *p* = 0.0017). Here, ΔΔCt was 0.78, corresponding to a 1.72-fold lower AKT2 transcript level in women with unexplained infertility (2^−ΔΔCt^ = 0.58). The standardized effect size was moderate (Cohen’s d = 0.59). This was the only variable in the dataset with an approximately normal distribution in both groups (Shapiro–Wilk *p* = 0.31), and the parametric and non-parametric tests agreed (Figure 2).

The difference persisted after adjustment. In a multivariable linear regression including group, age, body mass index, AMH, basal estradiol, and previous IVF attempts (*n* = 125), the adjusted between-group difference in ΔCt was 0.74 (95% CI 0.25 to 1.24; *p* = 0.004), corresponding to a 1.67-fold lower *AKT2* transcript level (95% CI 1.19 to 2.36). None of the covariates was independently associated with ΔCt (all *p* > 0.19). A sensitivity model substituting antral follicle count for AMH gave a comparable estimate (0.79; *p* = 0.002). The unadjusted and adjusted analyses therefore agree, and the group difference does not appear to be explained by the measured baseline characteristics.

### 3.3. Follicular Fluid Akt and Phospho-Akt

**Figure 3:**
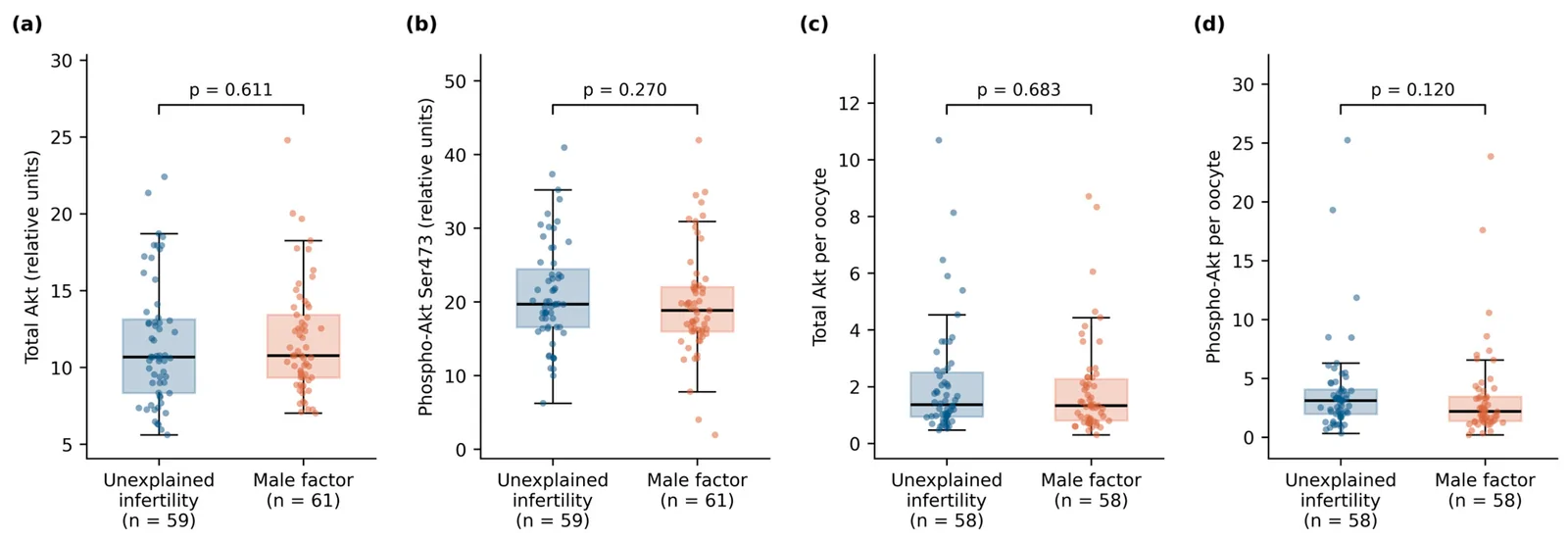
Follicular fluid Akt markers by group: (**a**) total Akt; (**b**) phospho-Akt (Ser473); (**c**) total Akt normalized per oocyte retrieved; (**d**) phospho-Akt normalized per oocyte retrieved. Values are expressed as relative units on assay-specific scales. Comparisons using Mann–Whitney U test.

Follicular fluid total Akt (11.55 ± 4.08 versus 11.75 ± 3.60 relative units; *p* = 0.78) and phospho-Akt (Ser473) (21.05 ± 7.14 versus 19.81 ± 7.24; *p* = 0.34) did not differ between groups. Normalizing to the number of oocytes retrieved did not change this (total Akt 2.08 ± 1.96 versus 1.91 ± 1.73, *p* = 0.68; phospho-Akt 3.89 ± 4.12 versus 3.38 ± 3.95, *p* = 0.12 with Mann–Whitney) (Figure 3). Because the pS473 and pan-Akt readouts are calibrated against a common kit standard rather than against purified isoform-specific protein, they are reported as relative units and are not interpretable as absolute Akt concentrations. For the same reason, a phospho/total ratio is not reported.

### 3.4. Embryological Outcomes

Mature oocyte rate (0.72 ± 0.25 versus 0.76 ± 0.18; *p* = 0.55), fertilization rate (44.6 ± 29.5% versus 42.2 ± 25.6%; *p* = 0.59), day 3 good-quality embryo rate (25.0 ± 25.8% versus 26.6 ± 23.8%; *p* = 0.59), and day 5 good-quality blastocyst rate (30.2 ± 25.8% versus 20.5 ± 18.5%; *p* = 0.25) were comparable (Table 3). Denominators declined across the sequence, most markedly at the blastocyst stage (16 and 18 women, respectively; Figure 1).

**Table 3:** PI3K/Akt markers and embryological outcomes.

Variable	UI	Male Factor	*t* Test *p*	Mann–Whitney *p*
Granulosa cell *AKT2* ΔCt	3.46 ± 1.47 (64)	2.68 ± 1.18 (64)	0.0011	0.0017
Follicular fluid total Akt, relative units	11.55 ± 4.08 (59)	11.75 ± 3.60 (61)	0.779	0.611
Follicular fluid phospho-Akt, relative units	21.05 ± 7.14 (59)	19.81 ± 7.24 (61)	0.345	0.270
Total Akt per oocyte	2.08 ± 1.96 (58)	1.91 ± 1.73 (58)	0.617	0.683
Phospho-Akt per oocyte	3.89 ± 4.12 (58)	3.38 ± 3.95 (58)	0.496	0.120
Mature oocyte rate	0.72 ± 0.25 (62)	0.76 ± 0.18 (61)	0.270	0.548
Fertilization rate, %	44.58 ± 29.53 (61)	42.22 ± 25.58 (61)	0.638	0.589
Day 3 good-quality embryo rate, %	25.04 ± 25.80 (58)	26.62 ± 23.81 (59)	0.732	0.587
Good-quality blastocyst rate, %	30.18 ± 25.83 (16)	20.54 ± 18.49 (18)	0.227	0.253

*AKT2*: ΔΔCt = 0.78; 2^−ΔΔCt^ = 0.58 (1.72-fold lower in UI); Cohen’s d = 0.59. Median [IQR] ΔCt: UI 3.37 [2.61–4.41] versus male factor 2.69 [1.82–3.34].

### 3.5. Exploratory Correlations

**Figure 4:**
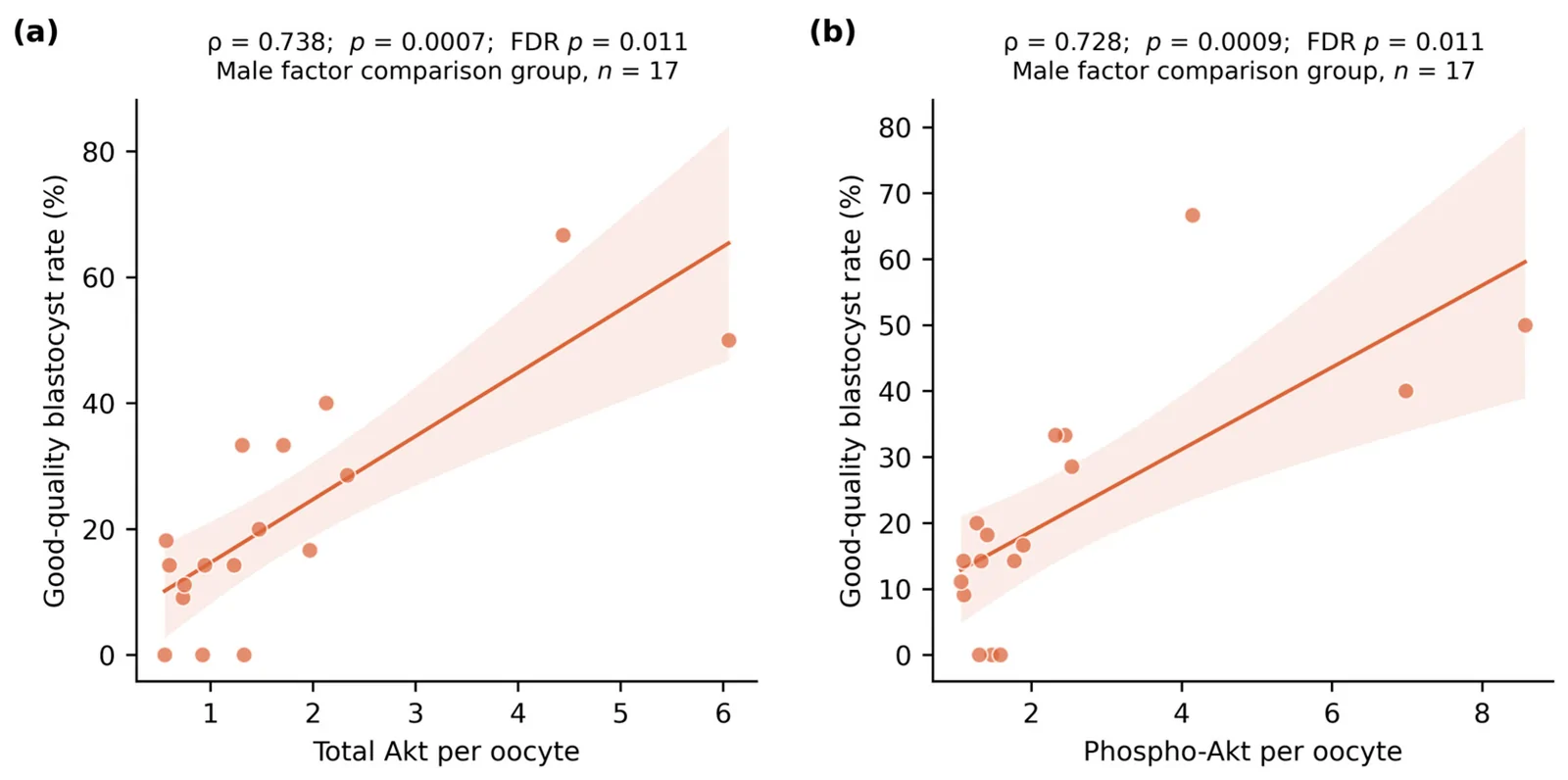
The two correlations surviving Benjamini–Hochberg adjustment, both in the male factor comparison group: (**a**) total Akt per oocyte and (**b**) phospho-Akt (Ser473) per oocyte versus the good-quality blastocyst rate. Each dot represents one participant (*n *= 17). The solid line is the least-squares regression fit and the shaded band is its 95% confidence interval. Spearman ρ, the raw *p* value and the FDR-adjusted *p* value are given above each panel.

Twenty-four Spearman correlations were computed (three markers × four endpoints × two groups; Table 4). Two survived FDR adjustment, both in the male factor comparison group and both at the blastocyst stage. These included total Akt per oocyte versus good-quality blastocyst rate (ρ = 0.738; raw *p* = 0.0007; FDR *p* = 0.011) and phospho-Akt per oocyte versus the same endpoint (ρ = 0.728; raw *p* = 0.0009; FDR *p* = 0.011), both with *n* = 17 (Figure 4). Bootstrap confidence intervals for these coefficients were wide (total Akt: 95% CI 0.33 to 0.92; phospho-Akt: 95% CI 0.30 to 0.91), reflecting the very small effective sample size. The point estimates should not be read as precise.

Five further associations reached nominal significance but did not survive adjustment: total Akt per oocyte with mature oocyte rate in both groups (UI ρ = 0.299, MF ρ = 0.295) and with blastocyst rate in the UI group (ρ = 0.532) as well as phospho-Akt per oocyte with mature oocyte rate in both groups (UI ρ = 0.261, MF ρ = 0.275). *AKT2* ΔCt did not correlate with any embryological endpoint in either group.

## 4. Discussion

The principal finding of this study is a 1.72-fold reduction in cumulus/granulosa cell *AKT2* transcript levels in women with unexplained infertility relative to women undergoing ICSI for isolated male factor infertility. The difference emerged in a cohort matched by design for age and stimulation protocol and found comparable on every conventional measure of ovarian reserve, ovarian response, and embryological performance. That contrast is the point: a measurable molecular difference within a patient group defined precisely by the absence of measurable abnormality.

Follicular fluid Akt and phospho-Akt concentrations, by contrast, were indistinguishable between groups by any formulation—raw or per-oocyte. This dissociation between transcript and protein findings is interpretable rather than contradictory. *AKT2* was measured in cumulus/granulosa cells, whereas Akt protein was measured in follicular fluid, a composite product of oocyte, granulosa, and theca compartments and of transudation from the systemic circulation [24]. Follicular fluid concentrations are therefore a diluted, compartmentally mixed readout of events within any single cell type. Transcript and protein abundance also correlate only moderately across human tissues [25]. A difference in cumulus cell mRNA need not manifest as a detectable difference in a pooled extracellular protein pool, and the negative protein result should not be read as contradicting the transcript result.

Several explanations can account for reduced *AKT2* transcript abundance alongside unchanged follicular fluid Akt and phospho-Akt, and they are not mutually exclusive. The first is isoform redundancy. The three Akt isoforms share substrate specificity to a considerable degree. In addition, compensatory *AKT1*—and possibly *AKT3*—activity could maintain total and phosphorylated Akt at the protein level despite lower *AKT2* message, which would also be consistent with the preserved embryological outcomes we observed. Because only *AKT2* was quantified, this study cannot test that possibility; isoform-resolved measurement is the obvious next step. The second is compartmental origin. Follicular fluid Akt derives from granulosa, theca, and oocyte compartments and from transudation across the basement membrane, so a change confined to the cumulus compartment may simply be diluted beyond detection in the pooled fluid. The third is post-transcriptional regulation. Transcript abundance and protein output are decoupled by translational control and protein turnover, and phosphorylation status is set by upstream kinase and phosphatase activity rather than by message level. The fourth explanation is the possibility that the reduction is a marker of an upstream process—altered gonadotropin responsiveness or an altered cumulus–oocyte signaling environment—rather than a lesion in Akt signaling itself. Distinguishing between these requires quantification of all three isoforms at both transcript and protein levels within the same cells, together with assessment of upstream and downstream pathway nodes.

The exploratory correlation analysis was, after appropriate adjustment for multiplicity, largely null. Only two associations survived FDR correction: follicular fluid Akt and phospho-Akt with blastocyst quality in the male factor comparison group. Both rest on 17 women, a subgroup small enough that the coefficients, although large, are imprecise. We consider these hypothesis generating at most. Notably, *AKT2* ΔCt showed no association with any embryological endpoint, so the group difference in transcript level is not explained by, and does not translate into, differences in laboratory performance within this cohort. Any inference that reduced *AKT2* expression impairs oocyte maturation or embryo development would go beyond what these data support.

The decision to sample cumulus and granulosa cells rather than the oocyte itself carries an interpretive cost that should not be left implicit. Transcript analysis of the oocyte destroys it, which cannot be justified within a clinical treatment cycle, whereas cumulus cells are discarded in the ordinary course of the procedure. What is gained in feasibility is lost in directness: a cumulus measurement reports the state of the somatic compartment, and any inference to the oocyte rests on the assumption that the coupling between the two compartments is preserved. If that coupling is itself altered in unexplained infertility—a possibility the present design cannot address—a cumulus marker would report something other than what it is assumed to report.

The comparability of embryological outcomes between UI and male factor cycles reproduces the findings of Alasmari et al. [26] and is itself relevant to the central problem. If the laboratory phenotype of UI is indistinguishable from that of couples with a defined cause, the etiological signal must be sought where current outcome measures do not resolve it. A reproducible transcript-level difference in the somatic cells immediately surrounding the oocyte is one place to look.

The comparison group is the principal interpretive constraint of this study and deserves emphasis rather than a passing mention. Women undergoing ICSI for isolated male factor infertility are not women of proven fertility; they are partners in infertile couples, and subfertility within a couple is not always cleanly attributable to one partner. Undetected female factors, shared lifestyle or environmental exposures, and the psychological and physiological context of prolonged infertility could all differ from a fertile population in ways this design cannot capture. Consequently, the observed difference establishes only that *AKT2* transcript abundance differs between two clinically defined infertile populations. It does not establish that the level seen in the comparison group represents the normal range, nor that the reduction is specific to unexplained infertility rather than reflecting characteristics of women in couples presenting with male factor infertility. A three-arm design including women of proven fertility—for example oocyte donors or women undergoing ICSI for non-infertility indications—would be required to resolve this, and we regard it as the necessary next study rather than an optional refinement.

Our approach sits within an established line of work using the cumulus compartment as a non-invasive readout. Transcriptomic profiling of cumulus cells has been proposed as a window onto oocyte maturation, with signatures differing between sibling oocytes of different maturity exposed to identical stimulation [27], and cumulus transcriptomic signatures have been related to live birth after ICSI [28]. Those studies pursued genome-wide discovery; the present study asks a narrower and more directed question about a single pathway component with a clear prior biological rationale. The two approaches are complementary, and the finding reported here would be a natural candidate for confirmation within a broader transcriptomic framework.

Two baseline variables differed nominally between the groups and deserve comment. Women with unexplained infertility had undergone fewer previous IVF attempts and had slightly higher basal estradiol. The direction of the first is clinically plausible since couples with a defined male factor may reach ICSI earlier and repeat it, but a difference of roughly 0.4 cycles is unlikely to bear on granulosa cell transcript levels. The estradiol difference, approximately 10 pg/mL within the normal early follicular range, is of a magnitude comparable to assay variability. Neither would survive a multiplicity correction applied across the fourteen baseline comparisons, and neither is plausibly related to the primary outcome.

It is worth stating plainly what a difference of this magnitude would and would not support clinically. A 1.7-fold difference in mean transcript level with a moderate standardized effect size describes a shift between two distributions that overlap substantially; it does not describe a threshold separating individual patients. On this evidence, *AKT2* could not be used to classify a given woman as having or lacking a signaling defect, and nothing here supports its use as a test. What the finding does support is a more specific research question than the one usually asked of unexplained infertility: not whether these women differ in some unspecified way, but whether a defined signaling pathway in a defined cell type is quantitatively altered—a question that can be answered.

### 4.1. Strengths and Limitations

Strengths include the prospective design, restriction to a single stimulation protocol and a narrow age band, a clinically well-characterized male factor comparison group whose female partners have no identified infertility factor, blinded sample coding, and complete AKT2 data for all 128 participants.

Limitations are substantial and constrain interpretation. First, a single pathway component was quantified against a single reference gene. Upstream (PI3K, PTEN) and downstream (FOXO3a, mTOR) nodes were not assessed, so pathway activation status was not determined. The present data speak only to AKT2 transcript abundance [29]. Second, protein measurement relied on ELISA in pooled follicular fluid without Western blot or immunocytochemical localization to specific follicular cell types. Third, clinical endpoints—implantation, clinical pregnancy, and live birth—were not available; all outcomes reported here are laboratory surrogates. Fourth, the blastocyst subgroup comprised only 16 and 18 women since most transfers were performed at cleavage stage, so blastocyst-stage estimates are imprecise. Fifth, the correlation analyses were exploratory and, after FDR adjustment, almost entirely null; the study was powered for the group comparison, not for these associations. Sixth, several methodological records from the original laboratory work are no longer retrievable. The instrument export files containing individual technical-replicate Ct values are unavailable, so the coefficient of variation between replicates cannot be computed retrospectively. RNA concentration and purity measurements for individual samples are likewise unavailable, and samples were assayed in single wells rather than in duplicate with ELISA. The reference-gene Ct values were also later than is typical for GAPDH, which is consistent with limited input RNA from small cumulus cell pellets and further limits the precision of the ΔCt estimates. Seventh, this was a single-center study in one population. Lastly, women in the comparison group were undergoing ICSI for male factor infertility and cannot be equated with women of proven natural fertility. The absence of a fertile reference arm means that the normal range of AKT2 expression in this cell population remains undefined, and residual confounding by unmeasured female-partner factors cannot be excluded despite adjustment for the measured covariates.

### 4.2. Future Directions

Confirming and extending this observation requires simultaneous quantification of several pathway nodes, cell type-resolved localization of total and phosphorylated protein, and linkage to clinical pregnancy and live birth rather than to laboratory surrogates. Cumulus-specific transcriptomic profiling would establish whether the reduction is restricted to *AKT2* or reflects coordinated pathway downregulation, and whether it marks a biologically distinct subgroup within the heterogeneous category of unexplained infertility.

## 5. Conclusions

Cumulus and granulosa cell AKT2 mRNA levels were approximately 1.7-fold lower in women with unexplained infertility than in women undergoing ICSI for isolated male factor infertility, and the difference persisted after adjustment for measured baseline characteristics. Ovarian reserve, ovarian response, embryological outcomes, and follicular fluid Akt and phospho-Akt protein levels were comparable between the groups, and AKT2 ΔCt did not correlate with any embryological endpoint. These data therefore describe a difference in transcript abundance, not a demonstrated alteration of PI3K/Akt pathway activity. In addition, because the comparison group comprised women from couples with male factor infertility rather than women of proven fertility, the difference cannot be attributed specifically to unexplained infertility. The finding is best regarded as a hypothesis-generating observation that requires confirmation against a fertile comparison group with isoform-resolved protein measurement in the same cells.

## Figures and Tables

**Table 1:** Primer sequences and characteristics for RT-qPCR.

Gene	Direction	Sequence (5′→3′)	Length (nt)	Tm (°C)	GC (%)
*AKT2*	Forward	CAGACGAGAGGGAGGAGTGGATG	23	63.7	60.9
*AKT2*	Reverse	CTGGGGGAGCCACACTTGTAGTC	23	64.6	60.9
*GAPDH*	Forward	CATGAGAAGTATGACAACAGCCT	23	58.5	43.5
*GAPDH*	Reverse	AGTCCTTCCACGATACCAAAGT	22	59.1	45.5

Amplification was performed with the Hibrigen PI3K/AKT gene expression analysis kit (catalog no. MG-AKT-01).

**Table 2:** Baseline and ovarian response characteristics (mean ± SD).

Variable	UI (*n* = 64)	Male Factor (*n* = 64)	*t* Test *p*	Mann–Whitney *p*
Age, years	29.9 ± 3.6	29.4 ± 3.7	0.222	0.268
Height, m	1.63 ± 0.06	1.64 ± 0.05	0.090	0.102
Weight, kg	65.91 ± 10.36	68.09 ± 11.08	0.251	0.386
Body mass index, kg/m^2^	24.93 ± 3.83	25.22 ± 3.99	0.667	0.684
Antral follicle count	8.48 ± 3.59 (62)	9.25 ± 3.61 (61)	0.243	0.197
Duration of infertility, years	3.90 ± 2.59	3.87 ± 2.56	0.945	0.990
Previous IVF attempts, *n*	1.53 ± 1.04	1.94 ± 1.22	0.045	0.031
Oocytes retrieved, *n*	8.63 ± 5.40 (62)	9.70 ± 5.54 (61)	0.278	0.180
FSH, mIU/mL	6.13 ± 1.95 (63)	6.19 ± 1.69	0.839	0.977
LH, mIU/mL	4.81 ± 3.04	4.39 ± 1.89 (63)	0.354	0.793
Estradiol, pg/mL	54.37 ± 29.16 (63)	44.84 ± 18.12 (62)	0.030	0.014
Prolactin, ng/mL	18.60 ± 12.38 (63)	22.54 ± 15.61	0.118	0.129
TSH, µIU/mL	1.75 ± 0.95	2.23 ± 2.30	0.127	0.193
AMH, ng/mL	3.86 ± 3.04	4.26 ± 3.62	0.498	0.339

Numbers in parentheses give *n* where data were missing. One prolactin entry was corrected from 642.63 to 64.263 ng/mL against the source record before analysis (data entry error).

**Table 4:** Exploratory Spearman correlations surviving or approaching significance, with Benjamini–Hochberg FDR adjustment (24 tests).

Group	Marker	Endpoint	*n*	ρ	Raw *p*	FDR *p*
MF	Total Akt/oocyte	Good-quality blastocyst %	17	0.738	0.0007	0.011
MF	Phospho-Akt/oocyte	Good-quality blastocyst %	17	0.728	0.0009	0.011
UI	Total Akt/oocyte	Mature oocyte rate	58	0.299	0.023	0.146
MF	Total Akt/oocyte	Mature oocyte rate	58	0.295	0.025	0.146
UI	Total Akt/oocyte	Good-quality blastocyst %	16	0.532	0.034	0.146
MF	Phospho-Akt/oocyte	Mature oocyte rate	58	0.275	0.036	0.146
UI	Phospho-Akt/oocyte	Mature oocyte rate	58	0.261	0.048	0.164

The remaining 17 correlations were non-significant before adjustment (all raw *p* > 0.05), including every correlation involving *AKT2* ΔCt. The full table is provided as Table S1.

## Data Availability

The de-identified dataset and the analysis script are available from the corresponding author on reasonable request, subject to institutional approval.

## Supplementary Materials

The following supporting information can be downloaded at: https://www.mdpi.com/article/10.3390/biomedicines14092108/s1, Table S1: Complete set of 24 exploratory Spearman correlations between PI3K/Akt markers and embryological endpoints, by group, with raw and Benjamini–Hochberg FDR-adjusted *p* values; File S1: STROBE checklist for case–control studies.
