# Supplementary material for: Reduced Granulosa Cell AKT2 mRNA Expression in Women with Unexplained Infertility: A Prospective Case–Control Study of ICSI Cycles
Source: Biomedicines. 2026 Sep 18;14(9):2108. doi: 10.3390/biomedicines14092108 (PMC13604382; doi:10.3390/biomedicines14092108)
Supplement: Supplementary file 1 [file biomedicines-14-02108-s001.zip › Biomedicines_Supplementary_Table S1.pdf]

biomedicines

MDPI

Supplementary Materials

Reduced Granulosa Cell AKT2 mRNA Expression in Women with Unexplained Infertility: A Prospective Case–Control Study of ICSI Cycles

Özgür Uzun, Belgin Devranoğlu, Enis Özkaya, Huri Demirci, Gülden Tunalı, Zehra Sezer, Fatma Beyza Sağ and Emine Elif Güzel Meydanlı

Table S1. Complete set of 24 exploratory Spearman correlations between PI3K/Akt markers and embryological endpoints, by group, with raw and Benjamini–Hochberg FDR-adjusted p values.

**Group**	**Marker**	**Endpoint**	***n***	**Spearman ϱ**	**Raw *p***	**FDR-adjusted *p***
Male factor comparison	*AKT2* ΔCt	Day-3 good-quality embryo rate	59	0.026	0.8459	0.8461
Male factor comparison	*AKT2* ΔCt	Fertilization rate	61	-0.121	0.3545	0.5977
Male factor comparison	*AKT2* ΔCt	Good-quality blastocyst rate	18	-0.103	0.6843	0.8212
Male factor comparison	*AKT2* ΔCt	Mature oocyte rate	61	0.083	0.5224	0.6966
Male factor comparison	Phospho-Akt per oocyte	Day-3 good-quality embryo rate	56	-0.045	0.7398	0.8455
Male factor comparison	Phospho-Akt per oocyte	Fertilization rate	58	-0.131	0.3259	0.5977
Male factor comparison	Phospho-Akt per oocyte	Good-quality blastocyst rate	17	0.728	0.0009	0.0110
Male factor comparison	Phospho-Akt per oocyte	Mature oocyte rate	58	0.275	0.0364	0.1457
Male factor comparison	Total Akt per oocyte	Day-3 good-quality embryo rate	56	0.027	0.8461	0.8461
Male factor comparison	Total Akt per oocyte	Fertilization rate	58	-0.062	0.6413	0.8100
Male factor comparison	Total Akt per oocyte	Good-quality blastocyst rate	17	0.738	0.0007	0.0110
Male factor comparison	Total Akt per oocyte	Mature oocyte rate	58	0.295	0.0248	0.1457
Unexplained infertility	*AKT2* ΔCt	Day-3 good-quality embryo rate	58	0.163	0.2215	0.4834
Unexplained infertility	*AKT2* ΔCt	Fertilization rate	61	0.029	0.8260	0.8461
Unexplained infertility	*AKT2* ΔCt	Good-quality blastocyst rate	16	0.194	0.4724	0.6669
Unexplained infertility	*AKT2* ΔCt	Mature oocyte rate	62	0.115	0.3736	0.5977
Unexplained infertility	Phospho-Akt per oocyte	Day-3 good-quality embryo rate	54	-0.110	0.4296	0.6443
Unexplained infertility	Phospho-Akt per oocyte	Fertilization rate	57	-0.222	0.0974	0.2850
Unexplained infertility	Phospho-Akt per oocyte	Good-quality blastocyst rate	16	0.380	0.1470	0.3528
Unexplained infertility	Phospho-Akt per oocyte	Mature oocyte rate	58	0.261	0.0478	0.1639
Unexplained infertility	Total Akt per oocyte	Day-3 good-quality embryo rate	54	-0.137	0.3236	0.5977
Unexplained infertility	Total Akt per oocyte	Fertilization rate	57	-0.216	0.1069	0.2850
Unexplained infertility	Total Akt per oocyte	Good-quality blastocyst rate	16	0.532	0.0340	0.1457
Unexplained infertility	Total Akt per oocyte	Mature oocyte rate	58	0.299	0.0225	0.1457

Correlations are ordered by group, marker and endpoint. FDR adjustment was applied across all 24 tests using the Benjamini–Hochberg procedure. Two associations remain significant after adjustment, both in the male factor group at the blastocyst stage. Denominators differ between endpoints because of available-case analysis; no values were imputed. Higher *AKT2* ΔCt denotes lower transcript abundance.
