# Supplementary material for: Reduced Granulosa Cell AKT2 mRNA Expression in Women with Unexplained Infertility: A Prospective Case–Control Study of ICSI Cycles
Source: Biomedicines. 2026 Sep 18;14(9):2108. doi: 10.3390/biomedicines14092108 (PMC13604382; doi:10.3390/biomedicines14092108)
Supplement: Supplementary file 1 [file biomedicines-14-02108-s001.zip › File_S1_STROBE_Checklist.pdf]

File S1. STROBE Checklist for Case–Control Studies

Manuscript: Reduced Granulosa Cell AKT2 mRNA Expression in Women with Unexplained Infertility: A Prospective Case–Control Study of ICSI Cycles

Corresponding author: Özgür Uzun

Item descriptions below are summarized. The authoritative wording of each item is available at https://www.strobe-statement.org. Page numbers should be inserted after the manuscript has been laid out in the journal template.

No.	Item	Where addressed	Page
1a	Study design indicated in title/abstract	Title and Abstract — "A Prospective Case–Control Study"; Abstract, Methods	
1b	Informative, balanced abstract summary	Abstract (Background/Objectives, Methods, Results, Conclusions)	
2	Scientific background and rationale	1. Introduction, paragraphs 1–4	
3	Prespecified objectives / hypotheses	1. Introduction, final paragraph	
4	Key elements of study design stated early	2.1. Study Design and Setting	
5	Setting, locations, dates of recruitment and data collection	2.1 — assisted reproductive technology unit, December 2020 – May 2021	
6a	Eligibility criteria; sources and methods of case ascertainment and control selection; rationale for choice	2.2. Participants — age 23–35, uniform GnRH antagonist protocol, exclusion criteria; comparison group = women undergoing ICSI for isolated male factor infertility; rationale and its limits stated in 2.2	
6b	Matching criteria and number of controls per case	2.2 — groups matched by design for age band and stimulation protocol; 64 cases, 64 comparison participants (1:1)	
7	Variables defined: outcomes, exposures, predictors, confounders, effect modifiers	2.4. Embryological Assessment and Outcome Definitions; 2.5. ELISA; 2.6. RT-qPCR	
8	Data sources and measurement methods for each variable; comparability across	2.3–2.6 — sample collection, ELISA (ab126433), RT-qPCR (Hibrigen MG-AKT-01), blinded sample coding; Table 1 (primer details)	

No.	Item	Where addressed	Page
	groups		
9	Efforts to address potential sources of bias	2.1 (blinded coding of samples); 2.2 (restriction to a single protocol and narrow age band); 4.1. Strengths and Limitations	
10	How study size was determined	2.2 — a priori calculation: d = 0.5, two-sided $\alpha = 0.05$, 80% power → 63 per group; 64 enrolled	
11	Handling of quantitative variables; groupings and rationale	2.7. Statistical Analysis; ΔCt and per-oocyte normalization defined in 2.5–2.6	
12a	Statistical methods, including confounder control	2.7 — Welch t-test and Mann–Whitney U reported in parallel; multivariable linear regression adjusting for age, BMI, AMH, basal estradiol and previous IVF attempts; Spearman correlation	
12b	Methods for examining subgroups and interactions	2.7; 3.5 — exploratory correlations by group, Benjamini–Hochberg FDR adjustment	
12c	Handling of missing data	2.7; Figure 1 — available-case analysis, no imputation; denominators stated for every variable	
12d	Matching of cases and controls addressed in analysis	2.2; 2.7 — matching by design (age band, stimulation protocol) rather than analytic matching	
12e	Sensitivity analyses	2.7; 3.2; 3.5 — parametric and non-parametric tests reported in parallel; sensitivity model substituting antral follicle count for AMH; bootstrap confidence intervals for correlation coefficients	
13a	Numbers at each stage: eligible, examined, included, analysed	3.1; Figure 1 (study flow)	
13b	Reasons for non-participation at each stage	Figure 1 — losses and reasons at each step	
13c	Flow diagram	Figure 1	
14a	Characteristics of participants; information on exposures and confounders	3.1; Table 2	

No.	Item	Where addressed	Page
14b	Number of participants with missing data per variable	Table 2 and Table 3 (n stated per row); Figure 1	
15	Numbers in each exposure category	Table 3; Table 4	
16a	Unadjusted and adjusted estimates with precision; confounders adjusted for and why	3.2–3.5; Tables 3 and 4 — unadjusted group means ± SD with Cohen's d; adjusted between-group difference with 95% CI from multivariable regression; Spearman ρ with raw and FDR-adjusted p and bootstrap 95% CI	
16b	Category boundaries where continuous variables were categorized	Not applicable — exposures analysed as continuous variables	
16c	Translation of relative risk into absolute risk where relevant	Not applicable — no risk estimates reported	
17	Other analyses performed (subgroups, interactions, sensitivity)	3.5; Table 4 — 24 exploratory correlations with FDR adjustment	
18	Key results with reference to study objectives	4. Discussion, opening paragraph; 5. Conclusions	
19	Limitations, sources of bias and imprecision, direction and magnitude	4.1. Strengths and Limitations	
20	Cautious overall interpretation considering objectives, limitations, multiplicity, other evidence	4. Discussion, paragraphs 2–4; 5. Conclusions	
21	Generalizability / external validity	4.1 — single-centre, one population; comparison group not of proven natural fertility	
22	Funding source and role of funders	Funding statement — Scientific Research Projects Coordination Unit of Istanbul University-Cerrahpasa, project TTU-2020-35474	

Reference: von Elm E, Altman DG, Egger M, Pocock SJ, Gøtzsche PC, Vandenbroucke JP; STROBE Initiative. The Strengthening the Reporting of Observational Studies in Epidemiology (STROBE) statement: guidelines for reporting observational studies. *Lancet* 2007;370:1453–1457.
